# Statins impact on epigenetics of tumor cells

**DOI:** 10.1186/2050-6511-13-S1-A64

**Published:** 2012-09-17

**Authors:** Murtaza Kulaksiz, Martin Hohenegger

**Affiliations:** 1 Institute of Pharmacology, Center for Physiology and Pharmacology, Medical University of Vienna, 1090 Vienna, Austria

## Background

Histones are basic proteins and are modified by diverse post-translation modifications such as acetylation, methylation, phosphorylation and ubiquitinylation. These epigenetic modifications are important regulatory processes in proliferation, survival, differentiation and motility. The epigenetic gene regulation occurs in DNA methylation at DNA level and histone modification or chromatin remodelling at protein level. Several enzymes, like DNA methyltransferases (DNMTs), histone methyltransferases (HMTs), histone demethylases (HDMs), histone acetyltransferases (HATs) and histone deacetylases (HDACs), are able to modify the chromatin. The histone modifications lead to alterations in chromatin and form heterochromatin or euchromatin, which activates or silences transcription. Statins are used successfully in the treatment of hypercholesterolemia and inhibit 3-hydroxy-3-methylglutaryl-CoA (HMG-CoA) reductase. HMG-CoA reductase is the rate-limiting enzyme of the mevalonate pathway. The synthesis of isoprenoids such as farnesylpyrophosphate (FPP) and geranylgeranylpyrophosphate (GGPP) is reduced by statins by inhibition of the HMG-CoA reductase. These isoprenoid intermediates are involved in post-translational modifications of Ras, Rho and Rac, which are typical for G proteins and have crucial roles in cancer cells.

## Methods

Western blot analysis was performed to quantify acetylation status of histones in SH-SY5Y and RD cell lines.

## Results

SH-SY5Y and RD cells were treated and incubated with increasing concentrations of simvastatin. The lysates were separated in a cytosolic and nuclear fraction. acetylated proteins were detected mainly in the nuclear fraction. Interestingly, the pattern of acetylation did not change very much upon statin exposure in the cytosol. However, in the nuclear fraction simvastatin extracts were acetylated to a greater extent.

## Conclusions

The data presented here suggest that simvastatin can affect histones and their post-translational modifications. Enhanced acetylation of nuclear proteins induced by simvastatin might be interpreted as an inhibition of a HDAC activity or/and as an increased HAT-mediated acetylation in SH-SY5Y and RD cells.

